# Expression levels of tumor necrosis factor-α and the corresponding receptors are correlated with trauma severity

**DOI:** 10.3892/ol.2014.2575

**Published:** 2014-09-29

**Authors:** CHANG LIU, JIANWEI TANG

**Affiliations:** Department of Emergency Surgery, Jinshan Hospital, Fudan University, Shanghai 200540, P.R. China

**Keywords:** trauma, tumor necrosis factor-α, tumor necrosis factor receptors, tumor necrosis factor receptor 1, tumor necrosis factor receptor 2, p55, p75, patient

## Abstract

This study investigated the plasma levels of tumor necrosis factor α (TNF-α) and the expression levels of TNF receptors (TNFRs) in patients with multiple trauma, together with the association between the levels of this cytokine and these cytokine receptors with the severity of traumatic injury. Blood samples were obtained from 60 multiple trauma patients at hospital admission (within 2 h of injury), and 6–8 h and 1–5 days after admission. The plasma levels of TNF-α and TNFR1/TNFR2 were detected using enzyme immunoassay. TNFR1 and TNFR2 expression levels on leukocytes, including neutrophils, lymphocytes and monocytes, were determined by flow cytometry. Clinical parameters were determined by injury severity score (ISS). At hospital admission, the plasma TNF-α and soluble TNFR levels in the trauma patients were elevated compared with those of healthy controls. Increased expression levels of TNFR1 and TNFR2 were also detected on leukocytes, particularly on lymphocytes and monocytes. The expression levels of the cytokine and the corresponding receptors were correlated with the ISS. TNF-α and TNFR expression levels remained significantly elevated for up to the third to fifth day following the traumatic injury. In the trauma patients, increased levels of TNF-α and TNFRs were correlated with the severity of traumatic injury in the early post-injury period, supporting the hypothesis that trauma-provoked organ dysfunction may be caused by an overwhelming auto-destructive inflammatory response.

## Introduction

Trauma may produce direct organ system injury or be accompanied by hemodynamic alterations, which may result in organ dysfunction/failure. Traumatic injury may also initiate an acute inflammatory response in injured tissues or organs, which may induce an uncontrolled systemic inflammatory response and result in multiple organ dysfunction syndrome (MODS), previously known as multiple organ failure or multisystem organ failure. In general, the acute or systematic inflammatory response is considered to be mediated through the interactions among cytokines, including the activation of tumor necrosis factor (TNF) and the corresponding receptors and neuroendocrine pathways (1–3).

TNF-α is a key cytokine involved in the generation of the acute inflammatory response (1,3). This inflammatory cytokine is primarily produced by immune cells, such as monocytes and macrophages, but a number of non-immune cell types, including fibroblasts, neurons, keratinocytes and smooth muscle cells, also produce TNF. TNF-α acts as a key intermediary in the local inflammatory immune response and is an acute-phase protein that initiates a cascade of cytokines. Furthermore, high levels of TNF result in increased vascular permeability, thereby recruiting macrophages and neutrophils to the site of injury and/or infection (2,3). The action of TNF-α is mediated via cell-surface TNF receptors (TNFRs) (4). Two distinct members of the TNFR family are currently recognized: TNFR1, also known as p55, and TNFR2, termed p75 (5–7). TNFR1 is expressed constitutively in the majority of cell types, whereas TNFR2 expression is restricted to hematopoietic cells and discriminates between the murine and human forms of TNF-α (8). TNFR2 expression has been shown to be induced by TNF-α, IL-1 and interferon-γ in rat primary astrocytes (9). The two receptors may function individually or synergistically to mediate the biological activity of TNF-α.

The plasma levels of TNF and the respective receptors are increased in response to severe trauma (10–13). By contrast, results for elevated TNF-α levels following trauma are commonly negative; low TNF-α levels have been reported to promote the remodeling or replacement of injured tissue by stimulating fibroblast growth (14–20). Additional beneficial functions of TNF-α include its involvement in the immune response to bacterial and certain fungal, viral and parasitic invasions, as well as in the necrosis of specific tumors (21). The present study aimed to investigate the plasma levels of TNF-α and the corresponding receptors, as well as the expression levels of TNFRs on leukocytes in the early phase following multiple traumatic injuries and up to five days intensive care. The objective was to analyze the time-dependent correlations between these immunological parameters and injury severity.

## Subjects and methods

### Patients

A total of 60 trauma patients (42 males and 18 females, aged 18 to 72 years) were included in the study. These patients were treated at the Department of Emergency Surgery, Jinshan Hospital, Fudan University (Shanghai, China) over a time period of three years (2008–2011). On hospital admission, the injury severity score (ISS) was recorded for the assessment of injury severity (11,18). Patients who suffered from chronic immune deficiency or who were pregnant were excluded from the study. As determined by the mean ISS, the patients were divided into the following three groups (n=20 patients per group): Low ISS (L-ISS; 9≤ ISS <16), medium ISS (M-ISS; 16≤ ISS <25) and high ISS (H-ISS; ISS ≥25). The control group included 20 healthy volunteers between the ages of 18 and 25 years. This study was conducted in accordance with the Declaration of Helsinki and with approval from the Ethics Committee of Jinshan Hospital, Fudan University. Written informed consent was obtained from all participants.

### Blood samples

Venous blood was collected from the patients and healthy volunteers, and was maintained in tubes with ethylenediaminetetraacetate anticoagulant. A volume of 10 ml blood was sampled from all patients at five defined time points. The first sample was obtained upon hospital admission by the emergency physician (usually within 2 h of injury) and the second sample was obtained 6–8 h after admission (or 8–10 h after trauma). Further blood samples were collected 24 h after admission, and then on days 3 and 5 post-admission. Following cooling centrifugation (20 min at 4°C and 1,500 × g), the plasma was aliquoted and frozen at −70°C in 250-μl aliquots for TNF-α and soluble TNFR1/TNFR2 (sTNFR1/sTNFR2) expression analysis.

### Measurement of TNF-α, sTNFR1 and sTNFR2 levels

The concentrations of TNF-α and the corresponding receptors in the plasma were determined by enzyme-linked immunosorbent assay (ELISA) techniques. The analyses were performed in duplicate using standardized, commercially available enzyme immunoassay kits according to the manufacturers’ instructions. A human TNF-α chemiluminescent ELISA kit (Thermo Fisher Scientific, Waltham, MA, USA) and sTNFR ELISA kits (Abcam, Cambridge, MA, USA) were employed. The sensitivities of the assays were <1 pg/ml for TNF-α and sTNFR1, and <5 pg/ml for sTNFR2. The control levels of the cytokine and the receptors were determined from the healthy volunteers.

### Flow cytometric detection of TNFRs in leukocytes

Whole blood samples were collected by standard syringe venipuncture upon hospital admission (within 2 h of injury) and mixed with anticoagulant (heparin, 10 IU/ml). Control samples were collected from matched healthy volunteers at similar time points in the day to when patient samples were collected. The cell samples were stained in the dark on ice for 60 min with affinity-purified rabbit polyclonal antibody for TNFR1 (1:500; Abcam), mouse monoclonal antibody for TNFR2 (MR2-1; 1:1,000; Abcam) or the appropriate controls (isotype or unstained). Antibody-binding was visualized with anti-rabbit or -mouse IgG conjugated to a fluorophore (60 min on ice) (Thermo Fisher Scientific). The samples were analyzed using a Gallios flow cytometer (Beckman Coulter, Inc., Brea, CA, USA) and CXP Analysis software (v2.1; Applied Cytometry Systems, Dinnington, UK). The expression levels of TNFRs in neutrophils, lymphocytes and monocytes were assessed, and are expressed as the mean fluorescence intensity following subtraction of the isotype control value.

### Statistical analysis

Differences among groups were compared using one-way or two-way analysis of variance (for degree of injury and leucocyte type) with Bonferroni’s post hoc analysis. The time-course changes in the expression levels of TNF-α and the corresponding receptors were analyzed by linear regression. The correlation coefficients (r) were calculated using Spearman’s rank test. P<0.05 was considered to indicate a statistically significant difference and data are expressed as the mean ± standard deviation. SPSS 11.0 (SPSS, Inc., Chicago, IL, USA) and GraphPad Prism 5 (GraphPad, La Jolla, CA, USA) were used for data analysis.

## Results

### Plasma TNF-α levels in trauma patients

**Figure 1:**
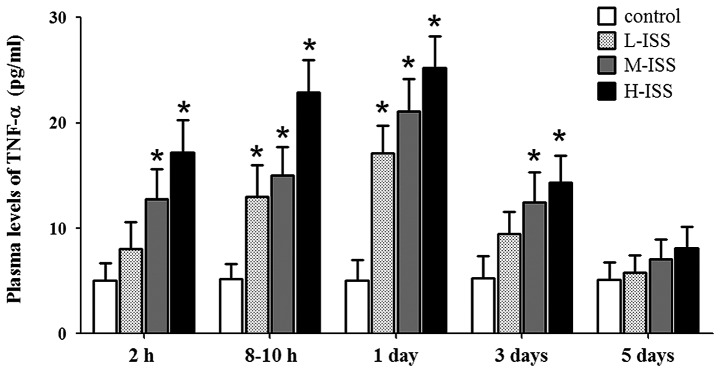
Time-course of plasma TNF-α levels in response to injury. Blood samples were collected at hospital admission (usually within 2 h of injury), and 6–8 h (8–10 h after trauma) and 1–5 days after admission. Plasma was analyzed for TNF-α levels using enzyme immunoassay. Traumatic injury resulted in an increase in plasma TNF-α levels, particularly in patients with high severity scores. Data are presented as the means ± SD, n=20 per group. ^*^P<0.05, compared with the healthy controls. TNF-α, tumor necrosis factor α; L/M/H-ISS, low/medium/high injury severity score.

Plasma TNF-α levels in all three trauma groups (L-ISS, M-ISS and H-ISS) were significantly elevated following injury (usually within 2 h trauma) as compared with the healthy controls (5.05 pg/ml in controls versus 8.07–17.23 pg/ml in patients; P<0.05; Fig. 1). Peak plasma TNF-α levels were detected 24 h after injury and the levels remained significantly elevated until up to the third day following trauma. At five days after injury, plasma TNF-α levels had gradually returned to the normal levels (Fig. 1). Furthermore, the TNF-α levels were significantly correlated with the severity of injury, as indicated by the ISS (r=0.78, P<0.0001).

### sTNFR plasma levels in patients with severe trauma

**Figure 2:**
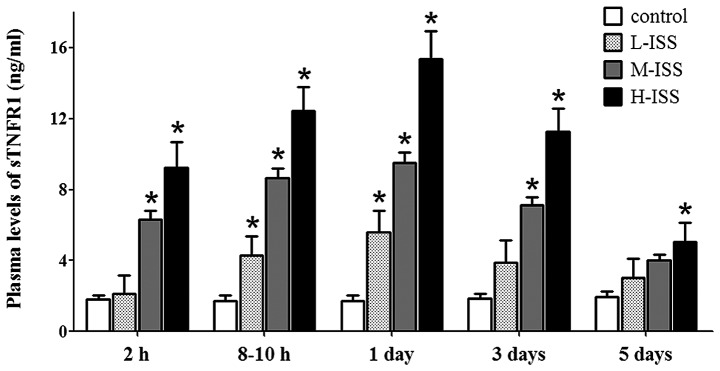
Plasma levels of sTNFR1 in patients with severe trauma. Traumatic injury upregulated the expression of the soluble receptor, with peak levels 1 day after trauma. Patients in the H-ISS group exhibited the highest sTNFR1 plasma levels at all five time points examined. Data are presented as the means ± SD, n=20 per group. ^*^P<0.01, compared with the healthy controls. sTNFR 1, soluble tumor necrosis factor receptor 1; L/M/H-ISS, low/medium/high injury severity score.

**Figure 3:**
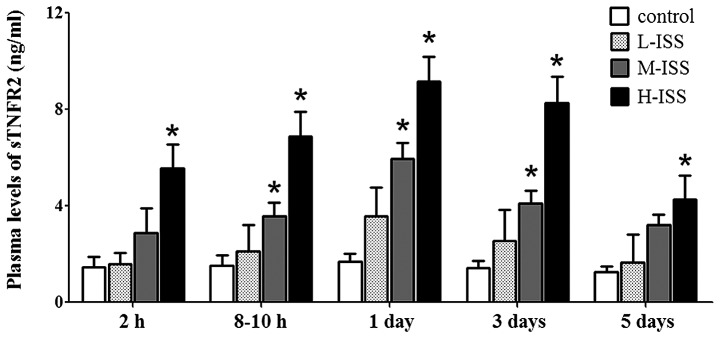
Plasma levels of sTNFR2 in patients with severe trauma. Elevated plasma levels of sTNFR2 were detected in the H-ISS and M-ISS groups, but not in the L-ISS group. Patients in the H-ISS group exhibited the highest sTNFR2 levels in the early phase of injury and even five days after trauma. Data are presented as the means ± SD, n = 20 per group. ^*^P<0.01, compared with the healthy controls. sTNFR2, soluble tumor necrosis factor receptor 2; L/M/H-ISS, low/medium/high injury severity score.

Within 2 h of injury, the sTNFR1 and sTNFR2 plasma levels were significantly elevated in all trauma groups compared with the normal controls (1.83±0.23 and 1.46±0.42 pg/ml for sTNFR1 and sTNFR2 in the controls, respectively). The patient group with the highest severity score (H-ISS group) exhibited the highest sTNFR1 and sTNFR2 plasma levels in the early phase following trauma. Elevated expression levels of soluble receptors were also observed 8–10 h after injury and 1, 3 and 5 days after trauma. sTNFR1 expression reached peak levels one day after trauma, which was gradually reduced and returned to normal five days after trauma (Fig. 2). Although increased levels of sTNFR2 were also detected in trauma patients compared with the controls, sTNFR2 levels were elevated for variable periods of time and were dependent on the severity of injury (Fig. 3). The statistical analysis suggested a significant correlation between the plasma levels of the sTNFRs and the severity of traumatic injury upon hospital admission (sTNFR1: r=0.89, P<0.0001 and sTNFR2: r=0.92, P<0.0001) as well as at the other four time points (data not shown).

### Expression levels of TNFRs on leukocytes

**Figure 4:**
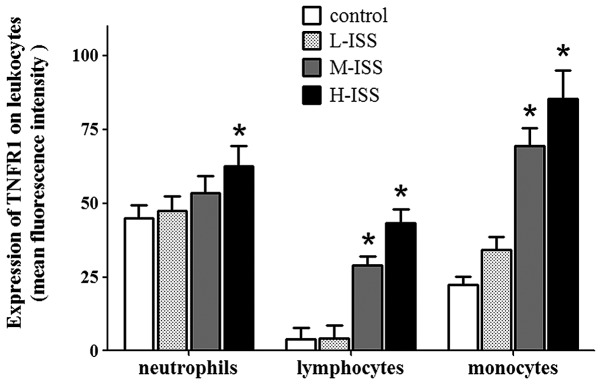
TNFR1 expression levels in leukocytes of patients with severe trauma. In the control patients, TNFR1 was highly expressed on neutrophils, moderately on monocytes and marginally on lymphocytes. Traumatic injury induced increased expression levels of the receptor, particularly on lymphocytes and monocytes. The blood samples were obtained within 2 h of injury, and surface expression levels of the receptor were assessed by flow cytometry. Values indicate mean fluorescence intensity and are presented as the mean ± SD, n=20 per group. ^*^P<0.01, compared with the healthy controls. TNFR1, tumor necrosis factor receptor 1; L/M/H-ISS, low/medium/high injury severity score.

**Figure 5:**
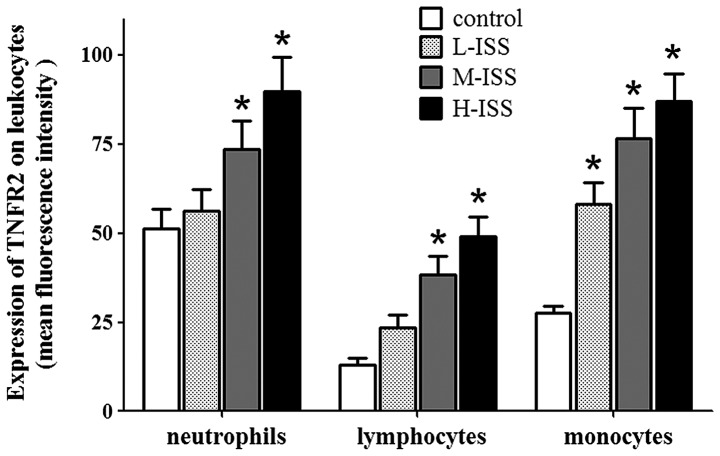
Flow cytometric analysis of TNFR2 expression levels in leukocytes. Monocytes were sensitive to traumatic injury and enhanced TNFR2 expression levels were detected in all trauma patients compared with the control individuals. Significantly increased receptor expression levels in neutrophils and lymphocytes were identified in patients with severity scores ≥16 (M-ISS and H-ISS groups). The blood samples were obtained within 2 h of injury, and surface TNFR2 expression levels are presented as the mean fluorescence intensity. Data are presented as the means ± SD, n=20. ^*^P<0.01, compared with the healthy controls. TNFR2, tumor necrosis factor receptor 2; L/M/H-ISS, low/medium/high injury severity score.

TNFR1 and TNFR2 were detected on leukocytes under control conditions, with high expression levels in neutrophils, moderate expression levels in monocytes and lower expression levels in lymphocytes. Traumatic injury resulted in enhanced TNFR1 and TNFR2 expression levels, with the highest expression levels in the H-ISS patient group. In particular, the injury-induced increase in expression levels of TNFRs was marked in monocytes and lymphocytes in the early phases following trauma. Furthermore, the increases in TNFR1 expression levels in monocytes and lymphocytes were significantly correlated with the severity of traumatic injury (monocytes: r=0.89, P<0.0001 and lymphocytes: r=0.93, P<0.0001; Fig. 4). A significant correlation between TNFR2 expression levels and injury severity was also detected in monocytes (r=0.89, P<0.0001) and lymphocytes (r=0.91, P<0.0001) (Fig. 5).

## Discussion

In the present study, the time-course changes in the expression levels of plasma TNF-α and the corresponding TNFR1 (p55) and TNFR2 (p75) receptors were examined in 60 trauma patients. Traumatic injury was found to elevate cytokine and soluble receptor plasma levels in the early phases of trauma, which remained elevated for up to the third to fifth day after the trauma. The TNFR expression levels on the surfaces of freshly harvested leukocytes in response to traumatic injury were also quantitatively analyzed; increased expression levels of TNFRs were found to be particularly marked in monocytes and lymphocytes. Notably, the plasma levels of the cytokine and the respective receptors, as well as the surface expression levels of the receptors on the leukocytes, were correlated with injury severity, as indicated by the ISS.

TNF-α is particularly important as part of the inflammatory response. This cytokine initiates the activation of several other cytokines and growth factors, as well as the recruitment of certain immune cells. TNF-α has been reported to be released more rapidly than other proinflammatory cytokines (22). In general, elevated TNF-α levels subsequent to trauma are harmful to the body (14–20), and increased TNF-α levels have been shown to induce central sensitization and hyperalgesia by increasing excitatory synaptic transmission (23). However, following traumatic injury to the brain, TNF-α exerts a neuroprotective role by contributing to neuroanatomical plasticity (24). In the present study, plasma TNF-α levels were detected to be elevated in all trauma patients immediately following trauma in comparison with healthy controls. Specifically, significant injury-induced elevation of TNF-α levels was detected up to three days subsequent to trauma. These results are consistent with those of Spielmann *et al* (20), in which a continuous and significant increase in TNF levels was identified in patients 4 h after trauma. In certain early studies, TNF-α was reported to be either unmeasurable (10,16,18) or not correlated with the severity of injury, and TNF-α is known as a sensitive parameter during sample processing. In the present study, plasma samples were collected immediately following blood withdrawal by cooling, centrifugation and freezing at −70°C. A highly sensitive ELISA kit was employed using enhanced chemiluminescence to detect TNF levels. Peak plasma TNF-α levels were detected 24 h after injury. Furthermore, the TNF-α levels remained significantly elevated for up to three days following trauma. At five days after injury, the plasma TNF-α levels gradually returned to normal. The data further suggest that the trauma-induced elevation in TNF-α levels was associated with the ISS.

Administration of TNF-α has observed to increase the expression levels of sTNFR1 and sTNFR2 in experimental conditions (25,26), suggesting a TNF-α-induced inflammatory response. TNF-α receptors may also be induced by direct damage to tissue, disturbed macro- and microcirculation with subsequent ischemia and/or reperfusion, and psychoneurological stimuli (27). In the present study, TNFRs were detected to be elevated on leukocytes as well as in the plasma of trauma patients, compared with healthy controls. Leukocytes are key anti-infectious agents in host defense, and neutrophils and monocytes are important inflammatory cells that mediate tissue damage. A flow cytometric method was established that enabled the quantitative evaluation of receptor expression levels on leukocytes in a single-cell suspension. The p55 and p75 receptors were found to be highly expressed on leukocytes, particularly on monocytes and lymphocytes, in response to traumatic injury. Notably, the increases in injury-induced TNFR expression levels were positively correlated with the severity of trauma. In the plasma, increased levels of TNFRs were also detected in the very early injury phase, and were associated with the severity of trauma. Furthermore, the expression levels of the two soluble receptors were correlated with the ISS in the early phase following trauma. These results confirm those of other studies, which demonstrated that the two receptor subtypes were involved in the inflammatory response. In early injury, sTNFR1 levels have been reported to be elevated directly following the accident, whereas sTNFR2 levels were increased after 24 h (20). The data from the present study indicated elevated sTNFR1 as well as sTNFR2 levels on admission to hospital, and receptor levels correlated significantly with the ISS. These data are consistent with those from other studies (28,29), in which soluble TNF-α receptors were shown to be more elevated in patients with lower survival rates. In addition, Froon *et al* (30) reported a significant elevation in sTNFR1 receptor expression levels during sepsis.

MODS is generally recognized as a predominant cause of mortality in trauma, systemic inflammatory response syndrome (SIRS) and a number of other critical illnesses. The systemic inflammatory response is rapidly followed in the majority of patients by a compensatory anti-inflammatory response, signifying an attempt to limit the SIRS response. Organ dysfunction is likely to ensue during an excessive inflammatory reaction. The patient is also at risk of opportunistic or secondary infection during an excessive anti-inflammatory response. Numerous potential humoral, cellular and exogenous mediators are involved in the pathogenesis of MODS/multiple organ failure, and various pathways that result in organ system dysfunction/damage (31). Among the potential mediators/pathways, TNF and the respective receptor signaling pathway may be critical in the pathogenesis of MODS. The data from the present study suggest that TNFRs are positively associated with the severity of injury. Therefore, inhibiting the activity of TNF-α and other cytokines as a therapy for trauma is of interest. Trials that aim to block, neutralize or remove the potential inflammatory mediators have shown success in several preclinical models of trauma, including spinal cord injury (32), brain injury (33) and liver injury (34), as well as in patients with chronic neurological dysfunction following stroke or traumatic brain injury (35).

In conclusion, the data from the present study demonstrate increased plasma levels of TNF-α and sTNFRs in response to severe traumatic injury. The study also provides invaluable data regarding TNFR expression in leukocytes, by quantitative assessment of the receptors on freshly harvested neutrophils, lymphocytes and monocytes using flow cytometry. The results highlight the potential correlation between TNFR expression levels and injury severity, supporting the hypothesis that an auto-destructive inflammatory response may cause trauma-initiated organ failure.

## References

[1] Lenz A, Franklin GA, Cheadle WG (2007). Systemic inflammation after trauma. Injury.

[2] Esposito E, Cuzzocrea S (2009). TNF-alpha as a therapeutic target in inflammatory diseases, ischemia-reperfusion injury and trauma. Curr Med Chem.

[3] Woodcock T, Morganti-Kossmann MC (2013). The role of markers of inflammation in traumatic brain injury. Front Neurol.

[4] Vandenabeele P, Declercq W, Beyaert R, Fiers W (1995). Two tumour necrosis factor receptors: structure and function. Trends Cell Biol.

[5] Loetscher H, Pan YC, Lahm HW, Gentz R, Brockhaus M, Tabuchi H, Lesslauer W (1990). Molecular cloning and expression of the human 55 kd tumor necrosis factor receptor. Cell.

[6] Smith CA, Davis T, Anderson D (1990). A receptor for tumor necrosis factor defines an unusual family of cellular and viral proteins. Science.

[7] Goodwin RG, Anderson D, Jerzy R (1991). Molecular cloning and expression of the type 1 and type 2 murine receptors for tumor necrosis factor. Mol Cell Biol.

[8] Tartaglia LA, Weber RF, Figari IS, Reynolds C, Palladino MA, Goeddel DV (1991). The two different receptors for tumor necrosis factor mediate distinct cellular responses. Proc Natl Acad Sci USA.

[9] Choi SJ, Lee KH, Park HS, Kim SK, Koh CM, Park JY (2005). Differential expression, shedding, cytokine regulation and function of TNFR1 and TNFR2 in human fetal astrocytes. Yonsei Med J.

[10] Cinat M, Waxman K, Vaziri ND, Daughters K, Yousefi S, Scannell G, Tominaga GT (1995). Soluble cytokine receptors and receptor antagonists are sequentially released after trauma. J Trauma.

[11] Ertel W, Keel M, Bonaccio M, Steckholzer U, Gallati H, Kenney JS, Trentz O (1995). Release of anti-inflammatory mediators after mechanical trauma correlates with severity of injury and clinical outcome. J Trauma.

[12] Stein DM, Lindell A, Murdock KR (2011). Relationship of serum and cerebrospinal fluid biomarkers with intracranial hypertension and cerebral hypoperfusion after severe traumatic brain injury. J Trauma.

[13] Dalgard CL, Cole JT, Kean WS (2012). The cytokine temporal profile in rat cortex after controlled cortical impact. Front Mol Neurosci.

[14] Hoch RC, Rodriguez R, Manning T, Bishop M, Mead P, Shoemaker WC, Abraham E (1993). Effects of accidental trauma on cytokine and endotoxin production. Crit Care Med.

[15] Rabinovici R, John R, Esser KM, Vernick J, Feuerstein G (1993). Serum tumor necrosis factor-alpha profile in trauma patients. J Trauma.

[16] Tan LR, Waxman K, Scannell G, Ioli G, Granger GA (1993). Trauma causes early release of soluble receptors for tumor necrosis factor. J Trauma.

[17] Cinat ME, Waxman K, Granger GA, Pearce W, Annas C, Daughters K (1994). Trauma causes sustained elevation of soluble tumor necrosis factor receptors. J Am Coll Surg.

[18] Svoboda P, Kantorová I, Ochmann J (1994). Dynamics of interleukin 1, 2, and 6 and tumor necrosis factor alpha in multiple trauma patients. J Trauma.

[19] Majetschak M, Flach R, Heukamp T (1997). Regulation of whole blood tumor necrosis factor production upon endotoxin stimulation after severe blunt trauma. J Trauma.

[20] Spielmann S, Kerner T, Ahlers O, Keh D, Gerlach M, Gerlach H (2001). Early detection of increased tumour necrosis factor alpha (TNFalpha) and soluble TNF receptor protein plasma levels after trauma reveals associations with the clinical course. Acta Anaesthesiol Scand.

[21] Zerey M, Burns JM, Kercher KW, Kuwada TS, Heniford BT (2006). Minimally invasive management of colon cancer. Surg Innov.

[22] Feldman AM (2008). TNF alpha - still a therapeutic target. Clin Transl Sci.

[23] Kawasaki Y, Zhang L, Cheng JK, Ji RR (2008). Cytokine mechanisms of central sensitization: distinct and overlapping role of interleukin-1beta, interleukin-6, and tumor necrosis factor-alpha in regulating synaptic and neuronal activity in the superficial spinal cord. J Neurosci.

[24] Oshima T, Lee S, Sato A, Oda S, Hirasawa H, Yamashita T (2009). TNF-alpha contributes to axonal sprouting and functional recovery following traumatic brain injury. Brain Res.

[25] Spinas GA, Keller U, Brockhaus M (1992). Release of soluble receptors for tumor necrosis factor (TNF) in relation to circulating TNF during experimental endotoxinemia. J Clin Invest.

[26] Van Zee KJ, Kohno T, Fischer E, Rock CS, Moldawer LL, Lowry SF (1992). Tumor necrosis factor soluble receptors circulate during experimental and clinical inflammation and can protect against excessive tumor necrosis factor alpha in vitro and in vivo. Proc Natl Acad Sci USA.

[27] Gerlach H, Gerlach M, Clauss M (1993). Relevance of tumour necrosis factor-alpha and interleukin-1-alpha in the pathogenesis of hypoxia-related organ failure. Eur J Anaesthesiol.

[28] Rogy MA, Coyle SM, Oldenburg HS (1994). Persistently elevated soluble tumor necrosis factor receptor and interleukin-1 receptor antagonist levels in critically ill patients. J Am Coll Surg.

[29] Pellegrini JD, Puyana JC, Lapchak PH, Kodys K, Miller-Graziano CL (1996). A membrane TNF-alpha/TNFR ratio correlates to MODS score and mortality. Shock.

[30] Froon AH, Bemelmans MH, Greve JW, van der Linden CJ, Buurman WA (1994). Increased plasma concentrations of soluble tumor necrosis factor receptors in sepsis syndrome: correlation with plasma creatinine values. Crit Care Med.

[31] Balk RA (2000). Pathogenesis and management of multiple organ dysfunction or failure in severe sepsis and septic shock. Crit Care Clin.

[32] Esposito E, Cuzzocrea S (2011). Anti-TNF therapy in the injured spinal cord. Trends Pharmacol Sci.

[33] Chio CC, Chang CH, Wang CC (2013). Etanercept attenuates traumatic brain injury in rats by reducing early microglial expression of tumor necrosis factor-α. BMC Neurosci.

[34] Shuh M, Bohorquez H, Loss GE, Cohen AJ (2013). Tumor necrosis factor-α: life and death of hepatocytes during liver ischemia/reperfusion injury. Ochsner J.

[35] Tobinick E, Kim NM, Reyzin G, Rodriguez-Romanacce H, DePuy V (2012). Selective TNF inhibition for chronic stroke and traumatic brain injury: an observational study involving 629 consecutive patients treated with perispinal etanercept. CNS Drugs.

